# Supplementary material for: The attitude of kidney transplant recipients towards elective arteriovenous fistula ligation
Source: PLoS One. 2020 Jul 2;15(7):e0234931. doi: 10.1371/journal.pone.0234931 (PMC7332306; doi:10.1371/journal.pone.0234931)
Supplement: S1 File — (DOCX) [file pone.0234931.s001.docx]

**English**

**Dear Sir or Madam,**

We kindly invite you to participate in the study which aims to evaluate the attitude of patients after a successful kidney transplantation to a functioning arteriovenous fistula, assess exercise capacity, as well as to obtain patients’ opinion on the maintenance of vascular access. There are no clear guidelines concerning the management of dialysis fistula after a successful kidney transplantation, and the issue of patients' approach has never been scrutinized in the literature either. Therefore, we kindly ask you to complete this anonymous survey. The results will be processed and used to improve health care of patients after kidney transplantation. By completing the survey, you agree to participate in the study.

**Please answer the following questions. Please underline the chosen answer.**

1. Gender: FEMALE MALE

2. Age: ..........

3. Height: .......... Body weight: ..........

4. Actual serum creatinine: ..........

5. When did you have a kidney transplantation? Please enter the date of transplantation: ..........

6. How and how long have you been on dialysis?

- HEMODIALYSIS
- PERITONEAL DIALYSIS

Please enter the number of months: ...................

7. What was the cause of renal failure?

- glomerulonephritis
- hypertensive nephropathy
- diabetic nephropathy
- interstitial kidney disease
- polycystic kidney disease
- other
- unknown

8. Do you have diabetes mellitus? YES / NO

9. Do you smoke cigarettes? YES / NO / NO, BUT I USED TO SMOKE

10. Do you have an active fistula? YES / NO

Please mark the localization of the fistula:

- FOREARM NEAR THE WRIST
- FOREARM NEAR THE ELBOW
- ARM

11. Have you ever considered fistula closure?

YES / NO / I DO NOT HAVE A CLEAR OPINION ON THIS MATTER

Why? ........................................................................................

12. Did any of the following factors influence your decision or opinion? Please underline the appropriate answer:

- my doctor's suggestion
- my family member's suggestion
- esthetical reasons

If your answer to question no. 10 was NO, please proceed to question 13.

If you were undergoing only peritoneal dialysis or hemodialysis through a catheter, please proceed to question 16.

13. Please underline the appropriate answer:

- the fistula was closed by a physician
- the fistula spontaneously thrombosed
- I have never had a dialysis fistula.

14. When did the ligation or thrombosis of your fistula take place? Please enter the date: ...................

15. Has your physical condition changed after the closure?

- YES, I FEEL BETTER
- YES, I FEEL WORSE
- NO

16. Do you have any of the following?

- heart failure YES / NO
- coronary artery disease YES / NO

17. Do any of the following symptoms apply to you?

- dyspnoe YES / NO
- lower extremity edema YES / NO
- fatigue YES / NO
- worse exercise tolerance YES / NO
- heart palpitations YES / NO

**Polish**

**Szanowni Państwo,**

Zapraszamy Państwa do wzięcia udziału w badaniu, którego celem jest ocena podejścia pacjentów po udanym przeszczepie nerki do funkcjonującej przetoki tętniczo-żylnej, przekrojowa ocena wydolności wysiłkowej, a także uzyskanie opinii na temat utrzymania dostępu naczyniowego. Nie ma jasnych wytycznych, jak postępować z przetoką dializacyjną po przeszczepie nerki, a podejście Pacjentów również nie było badane. Uprzejmie prosimy o wypełnienie ankiety, która jest całkowicie anonimowa. Wyniki zostaną opracowane i wykorzystane dla poprawy opieki nad pacjentami po przeszczepie nerki. Wypełniając ankietę wyrażają Państwo zgodę na udział w badaniu.

**Prosimy o udzielenie odpowiedzi na poniższe pytania. Prosimy o podkreślenie wybranej odpowiedzi.**

1. Płeć: KOBIETA MĘŻCZYZNA

2. Wiek: ..........

3. Wzrost: .......... Masa ciała: ..........

4. Stężenie kreatyniny: ……….

5. Kiedy miał/a Pan/Pani przeszczep nerki? Proszę podać datę przeszczepu: ..........

6. Jak i jak długo był/a Pan/Pani dializowany?

- HEMODIALIZA
- DIALIZA OTRZEWNOWA

Proszę podać liczbę miesięcy: ……………

7. Co było przyczyną niewydolności nerek?

- kłębuszkowe zapalenie nerek
- nefropatia nadciśnieniowa
- nefropatia cukrzycowa
- śródmiąższowe choroby nerek
- wielotorbielowate zwyrodnienie nerek
- inna
- nie wiadomo

8. Czy choruje Pan/Pani na cukrzycę? TAK / NIE

9. Czy pali Pan/Pani papierosy? TAK / NIE / NIE, ALE KIEDYŚ PALIŁEM/AM

10. Czy ma Pan/Pani czynną przetokę? TAK / NIE

Proszę zaznaczyć lokalizację przetoki:

- PRZEDRAMIĘ BLISKO NADGARSTKA
- PRZEDRAMIĘ BLISKO ŁOKCIA
- RAMIĘ

11. Czy zastanawiał/a się Pan/Pani nad zamknięciem przetoki? (jeśli posiadał/a Pan/Pani taką).

TAK / NIE / NIE POSIADAM SPRECYZOWANEJ OPINII NA TEN TEMAT

Dlaczego? ....................................................................................................................

12. Czy któryś z poniższych czynników wpłynął na Pani/Pana decyzję lub opinię? Proszę podkreślić właściwe:

- namawiał mnie do tego lekarz
- namawiała mnie do tego rodzina
- ze względów estetycznych

Jeśli był Pan/Pani dializowany otrzewnowo lub z wykorzystaniem cewnika, proszę przejść do pytania 16.

Jeśli odpowiedział/a Pan/Pani NIE na pytanie nr 10, proszę odpowiedzieć na pytania 13-15.

13.Proszę podkreślić właściwe:

- przetoka została zamknięta przez lekarza
- przetoka zakrzepła sama
- Nigdy nie posiadałem przetoki

14.Kiedy zamknięcie/zakrzepica przetoki miała miejsce? Proszę podać datę: ……………

15.Czy po ustaniu funkcji przetoki zmieniło się Pana/Pani samopoczucie?

- TAK, CZUJĘ SIĘ LEPIEJ
- TAK, CZUJĘ SIĘ GORZEJ
- NIE

16.Czy ma Pan/Pani rozpoznaną?

- niewydolność serca TAK / NIE
- chorobę niedokrwienną serca TAK / NIE

17.Czy poniżej wymienione dolegliwości Pana/Pani dotyczą?

- duszność TAK / NIE
- obrzęki kończyn dolnych (spuchnięte stopy, kostki) TAK / NIE
- zmęczenie, osłabienie TAK / NIE
- zmniejszona tolerancja wysiłku, łatwe męczenie się TAK / NIE
- kołatania serca TAK / NIE
