# Supplementary material for: The attitude of kidney transplant recipients towards elective arteriovenous fistula ligation
Source: PLoS One. 2020 Jul 2;15(7):e0234931. doi: 10.1371/journal.pone.0234931 (PMC7332306; doi:10.1371/journal.pone.0234931)
Supplement: Attachment — Submitted filename: Response to Reviewers.docx [file pone.0234931.s003.docx]

**Answer to Editor comments:**

**The manuscript was verified carefully to fulfill PLOS ONE requirements. Appropriate files are attached. The draft was checked and corrected by an official translator. The** questionnaire used in the study was attached as a supplementary file in the original and English version. Time frames when study was performed were added to the manuscript. Raw data set is attached and was also added into repository. Information is provided in Methods section. Ethics statement was moved according to instructions.

Answer to Reviewer 1 comments:

Thank you for your constructive remarks. The study was conducted in a limited number of patients from our center (about 30 %); however we got some interesting information that could be helpful in planning future trials. The approach to vascular access in a group of patients after transplantation is a topic of growing interest. Differences in the approach to vascular access in hemodialysis patients exist on country and international level, so there is a need to perform larger multicenter studies. The attitude to AVF closure in relation to time from transplantation was presented in revised manuscript.

Answer to Reviewer 2 comments:

Thank you for your positive evaluation and pertinent questions. The survey results have to be analyzed in concordance with the practice of the center. Our approach is to create AVFs distally, majority of renal transplant recipients in our center have forearm fistula. Due to lower flows a lot of them occlude within few months from transplantation. We also do not consider forearm AVFs as cardiotoxic, that could be not true in some cases. AVFs are also ligated rarely. I am sure, that in different center the survey results could be quite different. Aesthetic issues are actually very subjective and difficult to measure. However it was the most common reason to consider or to close AVF, it is actually a bit surprising. We did not find the difference in complaints profile in relation to vascular access location.

Answer to Reviewer 3 comments:

Thank you for your positive evaluation. Language editing was performed.
