# Supplementary material for: The attitude of kidney transplant recipients towards elective arteriovenous fistula ligation
Source: PLoS One. 2020 Jul 2;15(7):e0234931. doi: 10.1371/journal.pone.0234931 (PMC7332306; doi:10.1371/journal.pone.0234931)
Supplement: Attachment — Submitted filename: Response to Reviewers.docx [file pone.0234931.s004.docx]

**Dear Editor,**

**On behalf of all authors I would like to thank for positive evaluation of our manuscript. We appreciate the efforts of the Editorial Board and the Reviewers. We read the review carefully and revise the manuscript according to the suggestions done by Editor and Reviewers. Responses to all comments are included below.**

1. **Answer to Editor comments: The manuscript was** reviewed and revised **by native English speaker.**
2. Answer to Reviewer 1 comments: **Thank you very much for your favorable review.**
3. Answer to Reviewer 2 comments: **Thank you very much for your positive evaluation.**
4. Answer to Reviewer 3 comments: **The manuscript was** reviewed and revised **by native English speaker. Thank you very much for your constructive remarks.**

I hope that the manuscript will fulfill PLOS ONE publication criteria.

Yours sincerely,

Krzysztof Letachowicz
